# Using medical reality television as a technology-enhanced learning strategy to provide authentic patient care experiences during clinical placements: a case study research investigation

**DOI:** 10.1186/s12909-020-02432-7

**Published:** 2021-01-06

**Authors:** Fiona Osborne, Miles Harrison, James Fisher, Belinda Bateman

**Affiliations:** 1 School of Medical Education, Newcastle University, Cookson Building, Framlington Place, Newcastle Upon Tyne, NE2 4HH UK; 2 Education Department, Northumbria Healthcare NHS Foundation Trust, North Tyneside General Hospital, Rake Lane, North Shields, NE29 8NH UK; 3 Geriatric Medicine, Northumbria Specialist Emergency Care Hospital, Northumbria Way, Cramlington, Northumberland, NE23 6NZ UK

**Keywords:** Technology-enhanced learning, Emotion, Television, Distance learning, Online learning, Case study research, Coronavirus, Covid-19, Medical student, Education, Medical, Undergraduate

## Abstract

**Background:**

Over the last decade, the use of technology-enhanced learning (TEL) has rapidly expanded and diversified. Since the COVID-19 pandemic, there is a growing demand for distance and online learning strategies to support and even replace learning experiences previously afforded by clinical placements and clerkships. An intriguing but under-researched modality is the use of medical reality television to provide authentic experiences of patient care. This strategy does not feature in published medical educational literature, though promising research is emerging from other disciplines.

**Methodology:**

A programme of learning using medical reality television clips to facilitate case-based learning was developed according to the principles of ‘anchored instruction’, a technology-based educational theory. Clips were taken from the UK television show ’24 hours in A&E’. Medical students’ learning experiences were investigated using a qualitative approach addressing the following research questions:

- What is the perceived emotional experience of medical students when watching reality television in an educational context?

- How do medical students relate their experience of watching reality television in a formal educational setting to their perceived learning needs in the clinical environment?

A case study research methodology was adopted within the interpretivist paradigm_._ Data were triangulated from semi-structured interviews with students and non-participant observation of the teaching session. Field notes and transcripts were analysed through an inductive thematic analysis.

**Results:**

In response to the medical reality television, a diverse range of emotions were expressed including: excitement, amusement, concern, nervousness, sadness and joy. Students identified gaps in their clinical knowledge such as interpreting results, practical aspects of prescribing and end of life care. Key themes were increased student engagement and a promotion of holistic care practices.

**Discussion:**

Students perceived reality television as a highly realistic and relatable medium and an enjoyable, memorable way to contextualise learning from the classroom to real life, a finding mirrored in previous studies in other fields. The high degree of emotion expressed may explain the improved subjective memorability of the cases.

**Conclusion:**

Medical reality television offers a unique means of engaging students by providing authentic experiences of patient care and should be valued alongside other technology-enhanced learning strategies.

## Background

Over the last decade, the use of technology to enhance learning in undergraduate medicine has rapidly expanded and diversified [1, 2]. The youngest students have grown up in the digital age and expect learning to be technologically enhanced, convenient, personalised and relevant [3, 4]. Medical educators are increasingly using multimedia technology to facilitate learning, in particular where global events have necessitated a move to remote, distant and online learning [5, 6].

Yet at the heart of clinical education the interaction between students, professionals and patients has remained sacrosanct, as emphasised in the workplace learning discourses [7]. Bell’s 2009 study of students’ perception of interaction with ‘real’ patients suggests the value of verbal, visual and auditory experiences in providing meaningful experiences and an appreciation of perspective, context, and complexity [8]. However, opportunities for medical students to spend time in the clinical environment can be limited [9], particularly for smaller specialities [10, 11]. Increasing medical student numbers may compound competition to experience important but rare presentations [12]. In recent times, the COVID-19 pandemic has exacerbated existing tensions, leaving educators seeking technological solutions to replace curtailed or cancelled clinical placements [6, 13].

As an innovative Technology-Enhanced Learning modality, medical reality television may be worthy of exploration to increase students’ exposure to ‘real’ patients. Promising studies from business management and marketing report the benefits of using reality television to teach core principles [14, 15]. Yet, despite the longstanding use of television in medical teaching [16], there is a paucity of literature addressing the use of medical reality television - a Medline search by the authors did not yield any relevant published interventions or evaluations. Instead, previous studies have focused on the use of closed-circuit television [17], or fictional medical dramas, which though useful can be problematic in promoting potentially unhelpful professional stereotypes [3, 18–24].

A regional programme of teaching using reality television has been established in our UK centre for third year and final year medical students. Video footage was selected to portray acute, rare and sensitive presentations. The material was sourced from the television programme ’24 hours in A&E’. This is a UK based documentary presenting footage captured from 24 hour surveillance of the Emergency Department of a busy London hospital (filming initially at King’s College Hospital, latterly at St George’s Hospital).

The format of the sessions followed the principles of ‘anchored instruction’ as developed by Bransford and the Cognition Technology Group at Vanderbilt [25]. Anchored instruction is a technology-based learning theory, rooted in situated learning, which employs a multidimensional problem as an ‘anchor’ for students to solve by completing related, authentic tasks. It is described as facilitating students to develop ‘useful’ rather than ‘inert’ knowledge by developing their own perspective on a problem in context [25–27].

The impact of the programme on students’ learning was investigated through a qualitative study with two research questions:

- What is the perceived emotional experience of medical students when watching reality television in an educational context?

- How do medical students relate their experience of watching reality television in a formal educational setting to their perceived learning needs in the clinical environment?

The decision to explore the emotional experience of medical students derived from observations of intense emotional expression during the pilot intervention. It was considered prudent to investigate this further, given the recent ‘call to arms’ from prominent medical educators for more rigorous research into emotion as an important but neglected dimension of learning and clinical practice [28–31].

## Methods

Our research was based within the paradigm of interpretivism. From this vantage, reality is subjective and changing. Therefore, individuals ‘know’ and interpret their own reality, and there is no universal truth [32, 33]. Specifically, this investigation was framed from the perspective of social constructivism regarding knowledge as socially situated and meaning as constructed through social interaction [34, 35].

**Fig. 1:**
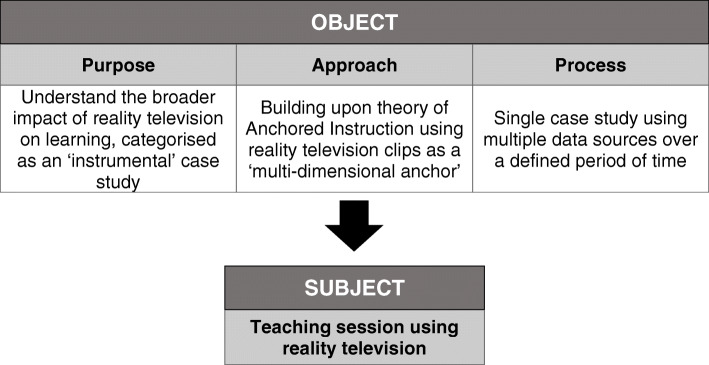
Case study approach used according to the Thomas and Meyers typology [37]

A case study research approach was used to gather and represent individuals’ interpretations to make collective sense of their experiences. The ‘contemporary phenomenon’ under investigation was a teaching session using reality television [36]. This was used as the ‘subject’ of examination as a key revealing case [36, 37]. The figure below (Fig. 1) demonstrates how this ‘subject’ was scrutinised according to the Thomas and Meyers typology of case-study research, the ‘object’ of enquiry [37, 38]. By adhering to these principles of a case-study research approach, the intention was to develop a deep and holistic understanding of the phenomenon [36].

### Ethical approval

Ethical approval was sought and obtained according to local health service and university requirements.

### Setting

This was a district-general ‘base-site’ of a UK medical school offering an integrated case-based curriculum.

### Participants

Participants were all final year students from the combined final year group of a five-year undergraduate programme/four-year post-graduate accelerated programme. The teaching session investigated was a timetabled mandatory session during the first week of a 16-week placement entitled ‘hospital-based practice’ covering acute medicine, surgery and emergency medicine. Most had experienced this method of teaching during a pilot in child health.

**Fig. 2:**
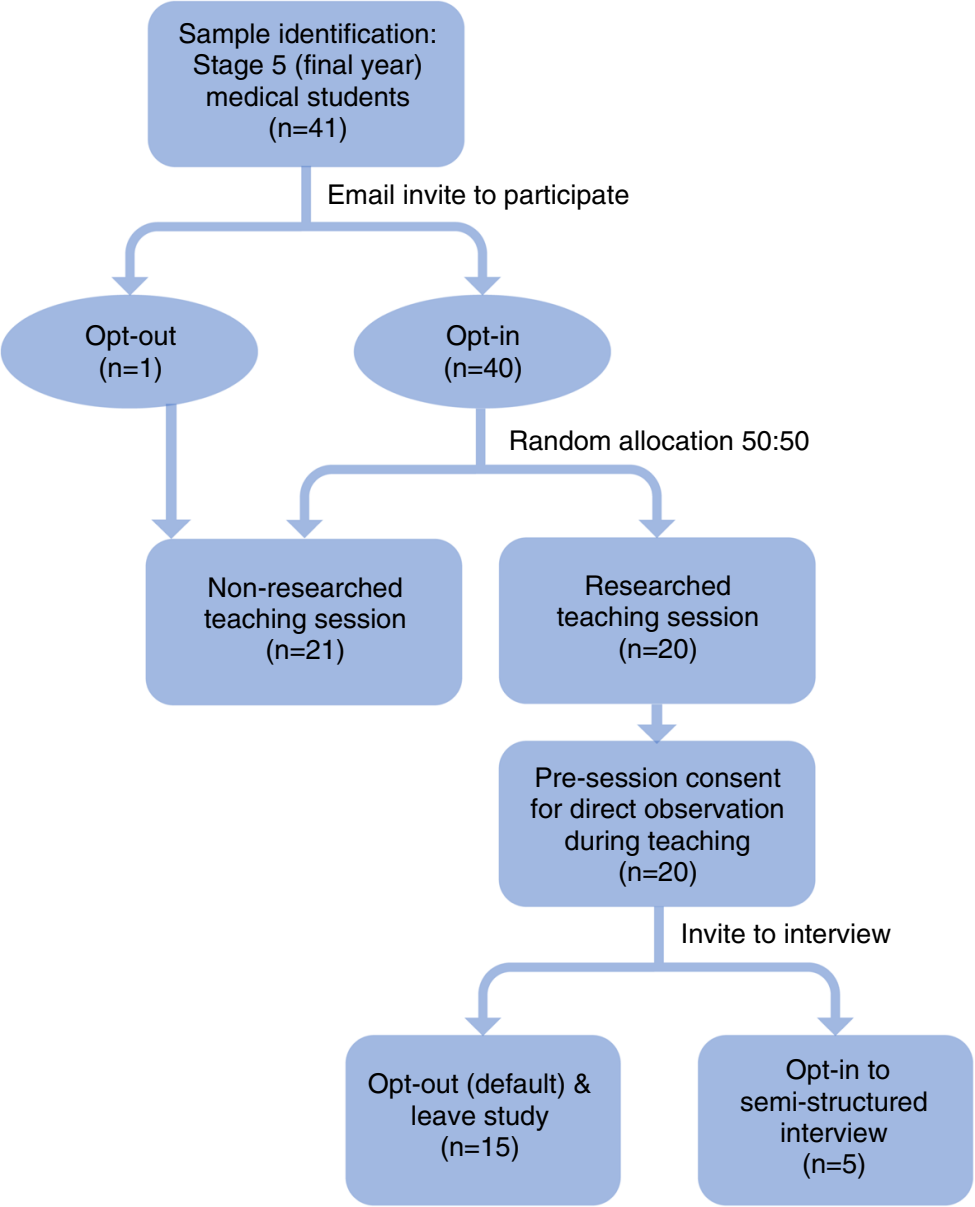
Outline of participant recruitment and involvement

An outline of participant recruitment and involvement in the study is outlined in the figure above (Fig. 2). The teaching session was scheduled twice in one day and only one session was subject to investigation to respect some students’ decision not to participate. The students were informed of the research study in advance by email and given a participant information sheet. All students were given the option of attending the ‘unresearched’ session. One student did so. The remaining students were randomly allocated between the groups by administration staff. Twenty students attended the ‘researched’ session and provided written consent on the day. All twenty were invited to participate in a semi-structured interview with the second author at the close of the session. Five students agreed (three females, two males), all undergraduate students with previous exposure to reality television teaching.

### Data collection

**Fig. 3:**
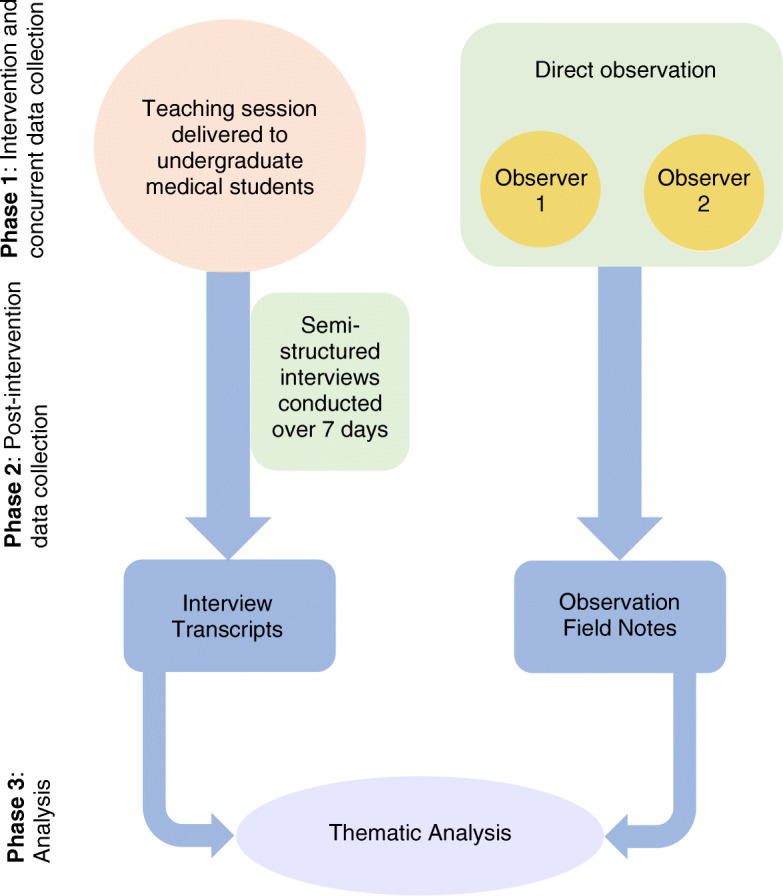
Study design for data collection and management

Data were collected from two sources in two consecutive phases. An overview of the process is outlined in the figure below (Fig. 3). Firstly, two experienced educationalists (second and fourth authors) engaged in non-participant observation [39] of the session, focusing on student activity. The second author was a teaching fellow in the base unit, a medical doctor with four years clinical experience enrolled on a medical education Masters. The fourth author was a consultant community paediatrician and experienced medical educationalist with responsibilities as associate sub-dean for the medical programme. The observers were reflexive about their role as researchers within the investigation when documenting the students’ reactions.

The second phase of data collection was through student participation in semi-structured interviews with the second author, supported by an agreed topic guide, during the 7 days after the session. Interviewees were encouraged to share their emotional experiences and reflections on their learning needs through the use of probing questions. The interviews were audio-recorded and transcribed.

### Data analysis

Field notes and interview transcripts were anonymised and then subjected to an inductive thematic analysis [40]*.* Coding was done on a line-by-line basis to establish data-driven inductive open codes. Subsequent coding then used the principles of ‘anchored instruction’ [25] as sensitising concepts to develop focused and axial codes where appropriate. Both data sets were independently coded by the first and second authors. When compared, there was a considerable degree of concordance with variations largely due to language or the extent to which codes were generalised. Consensus was reached on the coding strategy to be used, and thereafter the data was re-coded by both authors using the agreed, revised coding strategies. The final themes were agreed by all authors using the final focused and axial codes.

## Results

The results from field notes and interview transcripts have been integrated and are reported together. All names are pseudonyms.

### Judging the method

There was consensus that the video clips were useful and made the session more enjoyable and memorable. Students described feeling less passive and more actively engaged with the tasks and cases, a finding echoed in the field notes of the non-participant observers.

*‘I really enjoyed the session. Erm I, like, felt like an active participant which I don’t always feel like. It was, yeah, very useful’. Joanne.*

In contrast to paper cases which were described as ‘boring’, the video clips were considered to be realistic and the visual stimulus of seeing real patients in context gave the associated tasks meaning.

*‘You’re more inclined to answer the question properly … you felt more like you actively want, you obviously what you are gonna do isn’t gonna affect that patient but you felt more engaged to do it’. Vince.*

The video clips were not thought to be as useful as real patients because students could not interact with them. However, the session was compared to other simulation sessions as being a valuable ‘bridge’ to clinical practice in real settings.

### Developing as a clinician

During the video clips, the students described actively imagining themselves in the role of the doctors portrayed. This appeared to enable them to ‘test’ and identify gaps in their clinical knowledge, and reflect on how they would like to practise.

*“You have the little video of what’s happening, and in your head while the video is going on you’re thinking like right ok what sort of treatment is gonna be needed? What sort of management is this patient gonna need?” Milly.*

The students recognized gaps in their clinical skills, and identified areas they needed to revise including interpreting ECGs and radiographs. Joanne recalled that:

*“A lot of people didn’t even know to prescribe furosemide so it got like a lot of people thinking about that”. Joanne.*

A key area of learning described was that of managing the practicalities of patient death which the students described feeling unprepared for both practically, in terms of the paperwork required, but also emotionally. When discussing the patient who died, Bruce reflected:

*“Watching kind of the whole story unfold, hearing that she’s passed away mimicked a bit more than a paper case what it would be like in real life to kind of get to know someone, engage with them then to pass away. And then how you might reflect that in your documentation … it made me really reflect on how I might deal with that in the future.” Bruce*

### Emotions, empathy and connectivity

There was a high degree of emotion observed throughout the session and the students were candid in describing their experiences of elation, sadness, humour and tension. These reported emotions increased their empathy with the patients and families. One student described the session as an ‘emotional rollercoaster’ and that the emotional experience was a stimulus and a ‘hook’ to learning. A key example captured in the field notes was that the students appeared notably anxious when waiting to see if the patient with an unstable arrhythmia would be cardioverted.

*“It was quite tense I remember kind of, being like on the edge of my seat a lot.” Kirsty.*

The students also reflected on how their experience of intense emotions increased the memorability of the scenarios. Regarding the clip portraying a family member left alone in the Emergency Department after his relative is taken for life-saving surgery, Joanne said:

*“That was really sad. I think that was memorable because that was, ‘cause it was so sad...” Joanne.*

Certainly, the emotional experience of the students did appear to provoke a sense of empathy.

*“I remember us all just thinking he looked a bit kind of alone and hopeless and I really remember that kind of feeling it definitely brought out a lot of feelings with everyone” Bruce.*

Conclusively, the students described feeling ‘connected’ to the patients portrayed in the clips, particularly evident when one of the patients passed away.

*“I really remember a moment when we found that the patient … died and I think we were all quite invested in that because I remember everyone sat in silence at the end until it finished. No-one really said anything or did anything I think we all it kind of did affect people in that way they all felt quite connected to that session.” Bruce.*

This emotional experience appeared to mirror what the students might experience in the case of a death of a patient they knew well in clinical practice.

### Practising holistic care

As well as learning the principles of acute management of the patients portrayed, the students also reported learning about the ‘human’ aspects of caring for patients and their families. In particular, observing the realities of practice under pressure appeared to prompt the students to reflect on their person-centred communication skills. One clip sparking particular interest portrayed the attending doctor break ‘bad news’ to a patient’s family member in an open triage area.

*“It was one of the doctors had a conversation with one of the patient’s family like in the middle of the department and it was like a hard talk conversation, it was difficult communication and I thought that was done badly, and it was just like watching that on video made me cringe a little bit and I was like oh I would definitely never do that.” Milly.*

The insight this clip provided on the family experience of illness appeared particularly powerful in promoting compassionate care. Joanne reflected:

*“It also made you think about other things like the patient’s perspective, the family’s perspective so it was like, just yeah, hit multiple targets …” Joanne.*

Vince agreed:

*“It brought back being sort of you know how important it is just to have that little bit of a human touch like at the end of the day … just that little reassuring word or, just that something it has a massive impact on someone”. Vince.*

The narratives provided by the clips appeared to emphasise some students’ resolve to practise holistic care.

### An experience shared

An unexpected finding from the study was the extent to which the learning from reality television became a social experience of shared learning. The session was designed with a mix of group discussion and individual working on clinical tasks to facilitate development of their own perspective of the problem in context. However, the students chose to work collaboratively on the tasks and were acutely aware of others’ responses, and those of the tutors. Interestingly, one student reflected on her dissimilarity from her colleagues in responding to one of the patients’ deaths:

*“But then seeing everyone else get upset I was like awww no this is quite sad, maybe I should be sad too. [Laugh] but it didn’t affect me as much.” Kirsty.*

The group response potentially helped to ‘normalise’ the expression of emotion, particularly given that one student noted that one of the tutors cried. Another aspect of shared learning was the ‘tutors’ anecdotes. In one of the tasks about documenting deaths, the tutor shared their own practice of writing in the notes ‘may their soul rest forever in peace’. This was a shared point of discussion during the interview. Bruce described how he hoped to adopt this practice:

*“It’s only a small thing but to write something to make you feel better about the fact that you kind of closed the book on their life … in real life it’s definitely something I would write.” Bruce.*

## Discussion

### Reality television as a learning modality

This investigation found similar results to studies in business management, where students described the learning as enjoyable, realistic, relatable and relevant [15, 41]. Considering the concept of ‘realism’, students considered the session good preparation for clinical practice and, like mannequin-based acute simulation teaching, deemed it a ‘bridge’ to real clinical practice. This finding is consistent with the call for a broader understanding of the concept of ‘fidelity’ within medical education, where providing ‘real-life cues’ is considered as, if not more, important than aiming to recreate reality using expensive simulation technology [42, 43]. Conceivably, reality television should be valued alongside other simulation modalities as a low cost, low technology solution for providing medical students with authentic stimuli relevant to clinical practice.

### Understanding of emotion in medical student learning

There is recognition that medical training involves a diverse range of emotional experiences both inside and outside the classroom [28, 29, 44, 45]. There is a drive to better understand the emotional dimension of medical education, particularly given the growing evidence of the role played by emotion in learning [29, 31, 45, 46]. Within this educational setting, the use of clips from reality television did appear to provoke strong emotions within participants, which they described as promoting engagement and memorability of the subject matter.

However, the nature of this relationship merits further investigation. Interestingly, the use of short film clips is an established psychological experimental technique to ‘induce’ particular emotions in subjects [46]. Could it be the ‘story telling’ aspect of reality television programmes, produced and edited to ‘entertain’, which fosters this emotional experience? Moreover, should the provocation of strong emotions be an aim in itself? Taylor et al. criticised the deliberate manipulation of students’ emotions in a lecture setting, where a mistruth was told to induce stress and promote an understanding of the subject material (breaking bad news) [47, 48]. Arguably, the use of reality television, designed to entertain, could be considered a similar method of provoking students’ emotions unnecessarily or unfairly. Yet, as McConnell and Eva contend, doctors need to learn to function in a variety of emotional states [45]. Perhaps the ‘reality’ of reality television helps contextualise emotions, and therefore more closely simulates clinical practice than unedited film footage. Witnessing the tutors experience these emotions may also help overcome the problem of the ‘hidden curriculum’ promoting the unhealthy idea that doctors should learn to distance themselves from both their patients and their own emotions [49, 50].

### Strengths and limitations of the study

The study provides insight into previously unchartered terrain within medical education – that of using reality television as an educational tool. The novelty of the research subject and intervention is supported by the theoretical underpinnings of ‘anchored instruction’ as an established educational approach [25]. Considering the research design, the credibility of this case study approach is strengthened by triangulation. Elements included are data triangulation (both tutor and student as sources), methodological triangulation (two data collection methods) and investigator triangulation (non-participant observation conducted by two researchers) [51]. The development of robust themes was supported by ‘double-coding’ of all data by the first and second authors [52].

A limitation of the study relates to sampling. Considering the participants undergoing ‘observation’, a sample of suitable size and diversity was achieved through randomisation of the purposively selected final year student group. However, one student chose not to participate, meaning their perhaps unique perspective was lost. A relatively small sample of five students volunteered to be interviewed. This conceivably led to a reporting bias [53]. Another important limitation was the short window for data collection and analysis that prohibited an iterative approach. Interviews were all conducted over four days and analysis took place after data collection. There was no opportunity to pursue emerging concepts during the interviews nor to undertake further data collection enabling thematic saturation. As Braun and Clarke suggest, a pragmatic ‘in-situ’ decision was made with regard to the final sample size during data collection and analysis [54]. Having been immersed in the data that was obtained, it was felt to be sufficiently rich and complex to enable the research questions to be answered, despite our inability to achieve data saturation. We do however acknowledge that this strategy may mean that some students’ opinions and views remain unheard and unknown.

## Conclusion

Medical reality television may offer a unique means of engaging students by providing authentic experiences of patient care and should be valued alongside other technology enhanced learning strategies. Our findings suggest that when used as an adjunct to case-based learning, medical reality television supports medical students to identify their learning needs in the clinical environment and may help promote holistic care practices. This may in part be due to increased emotional engagement with case materials. Further research is required to determine the transferability of the findings to different healthcare settings and student groups and to explore the use of medical reality television in different learning modalities, for example when used as part of remote online learning or self-directed study.

## Data Availability

The datasets generated and analysed during the current study are not publicly available in order to preserve individual privacy. Data is available from the corresponding author on reasonable request.

## Abbreviation

TEL: Technology-enhanced learning
